# The Relationship Between Children's Birth Time and Short Stature

**DOI:** 10.3389/fped.2021.766448

**Published:** 2022-02-04

**Authors:** Shuo Wang, Na Shao, Yiyi Ding, Hong Cai, Runmei Zou, Cheng Wang

**Affiliations:** ^1^Department of Neonatology, Xiangya Hospital, Central South University, Changsha, China; ^2^Department of Pediatrics, The First People's Hospital of Changde City, Changde, China; ^3^Department of Pediatric Cardiovasology, Children's Medical Center, The Second Xiangya Hospital, Central South University, Changsha, China

**Keywords:** birth time, children, season, short stature, smooth curve fitting

## Abstract

**Background:**

There are few current reports on the relationship between time of birth and short stature in children. Therefore, we aimed to investigate whether there is an association between time of birth and short stature in children.

**Materials and Methods:**

In this study, basic information was collected from 462 children aged 2–14 years old. We collected data on gender, height, height standard deviation score (SDS), weight, body mass index (BMI), serum 25(OH)D levels, date of birth, and whether the above children were short stature. Demographic description, univariate analysis, multivariate logistic regression analysis, smooth curve fitting, and threshold effects were used to explore possible linear or non-linear relationships between children's birth time and short stature.

**Results:**

The mean age of the 462 children was 9.76 ± 3.10 years old, and 52.16% were male. A total of 129 (27.92%) children were defined as 25(OH)D insufficiency, including 107 (38.91%) in the short stature group and 22 (11.76%) in the normal stature group. Fully adjusted logistic regression showed that the risk of short stature was reduced by 56.5% in children born in summer compared with spring (*P* < 0.05) [odds ratio (OR): 0.435, 95% confidence interval (CI): 0.197–0.959]. A non-linear relationship was found between “sequential day of the year” and short stature from the 1st to the 250th day of the year, the risk of short stature in children is reduced by 0.6% for each day that passes (*P* = 0.002) (OR: 0.994, 95% CI: 0.990–0.998), and from the 250th to the 365th day of the year, the risk of short stature in children was increased by 0.8% for each day that passed (*P* = 0.008) (OR: 1.008, 95% CI: 1.001–1.025).

**Conclusions:**

Children born in summer have a lower risk of short stature than spring. For children born before the 250th day of the year, “sequential day of the year” was negatively associated with short stature, and for children born after the 250th day, “sequential day of the year” was positively associated with short stature.

## Introduction

Children's short stature (1) refers to a height two standard deviations (SD, third percentile) lower than the normal height of the normal population with the same living environment and individuals of the same race, gender, and age. Children's short stature is caused by many factors that may be related to various acute and chronic diseases, such as growth hormone deficiency, family heredity, multiple pituitary hormone deficiency, small gestational age, thyroid dysfunction, malnutrition, and pituitary tumors. Previous studies show that the etiology of certain diseases may be associated with seasonal changes. Palaniswamy et al. (2) indicate that gender, season, latitude, alcohol consumption, and physical activity are the main factors affecting the concentration of 25(OH)D. Risk factors for low 25(OH)D status include low-day exposure, living in the northern latitudes, obesity, high waist circumference, low physical activity, and unhealthy diet. Differences in birth time are accompanied by different environmental factors, such as temperature, lighting time and intensity, and length of day and night. Waldie et al. (3) deem that during the last 3 months of pregnancy, when the sunshine reaches its peak, the growth rate was the fastest during childhood and adolescence, suggesting that the occurrence of short stature may be affected by the birth rhythm. Xu et al. (4) suggest that infants in Shanghai city of China had obvious seasonal growth, and their height increased rapidly in spring and summer. The body weight and body mass index (BMI) increased rapidly in autumn and winter. Barker proposed the hypothesis of “fetal origin of adult disease” in 1993 (5), establishing that maternal malnutrition may lead to an increased risk of cardiovascular disease and altered metabolic status in the offspring. Mary et al. (6) conclude that geographic climate change with the change of seasons plays an important role in human health and disease. It is reported (7, 8) that maternal exposure to many environmental variables during pregnancy is associated with an increased risk of disease in children when they grow up. For example, insufficient daylight hours (3) and higher moderation (9) can increase the risk of disease in offspring. Felix et al. (10) report that children born in summer had higher average birth weight and higher adult height compared with those born in other seasons, and the opposite was true in winter. In this study, we hypothesized that there is an association between the time of birth and short stature in children. We collected relative data in our clinical work to verify our conjecture and tried to analyze the potential correlates that may lead to children's short stature.

## Methods

### Study Population

This study is a case-control study to explore the relationship between children's birth time and short stature. We collected 2- to 14-year-old children (*n* = 275) who were diagnosed with short stature in the Children's Endocrinology Department of The First People's Hospital of Changde City from January 1, 2019 to June 30, 2019. Children with normal stature (*n* = 187) who were given a health examination at the hospital at the same time were selected for comparison.

This study was approved by the ethics committee of The First People's Hospital of Changde City(2020-019-01).

### Inclusion and Exclusion Criteria

According to the published guidelines (1), children attending a pediatric endocrinology clinic who were −2 SDS from the mean height of the normal population or below the third percentile (−1.88 SDS) were included in the short stature group. The rest of the children in the normal stature group were all healthy children whose stature did not match the standard of short stature.

Children with incomplete data, a history of acute or chronic illness, treated with recombinant human growth hormone, or small for gestational age were excluded.

### Variables

All children's age, gender, height, height SDS, weight, BMI, serum 25(OH)D, and birth time were recorded. Information on the birth dates of the above children were classified as continuous or categorical variables. The detailed process is described as follows: the season to which the birth date belongs is considered to be a categorical variable: Spring (March-April-May), Summer (June-July-August), Autumn (September-October-November), Winter (December-January-February). The date of birth is considered as a continuous variable, sequential day of the year. We considered whether children met the short stature criteria as a categorical variable.

We classified the collected serum 25(OH)D values with a cutoff of 20 ng/ml by referring to another study (11): When serum 25(OH)D < 20 mg/ml defined as insufficiency. When serum 25(OH)D ≥ 20 ng/ml defined as sufficiency.

### Statistical Analysis

If the continuous variable was normally distributed, it was expressed as mean ± SD and vice versa as the medium (min, max). Categorical variables were expressed in frequency or as a percentage. χ^2^ (categorical variables), Student's *t-*test (normal distribution), or Mann–Whitney *U*-test (skewed distribution) were utilized to analyze differences between short or normal stature groups (clinical cut point). We used multiple logistic regression to analyze the possible association between season of birth and short stature and constructed three models to illustrate the stability of this relationship: Model I adjust for none; Model II adjust for gender and age (smooth); and Model III adjust for gender, age (smooth), weight (smooth) and 25(OH)D. To address non-linearity of children's birth time and short stature, a generalized additive model and smooth curve fitting (penalized spline method) were conducted. If non-linearity was detected, we first calculated the inflection point using a recursive algorithm and then constructed a two-piecewise logistic regression on both sides of the inflection point. We determined the best fit model based on the *P*-values for the log likelihood ratio test. All the analyses were performed with the statistical software packages R (version 3.4.3) (http://www.R-project.org, The R Foundation) and EmpowerStats (http://www.empowerstats.com, X&Y Solutions, Inc, Boston, MA). *P*-values < 0.05 (two-sided) were considered statistically significant.

## Results

**Figure 1:**
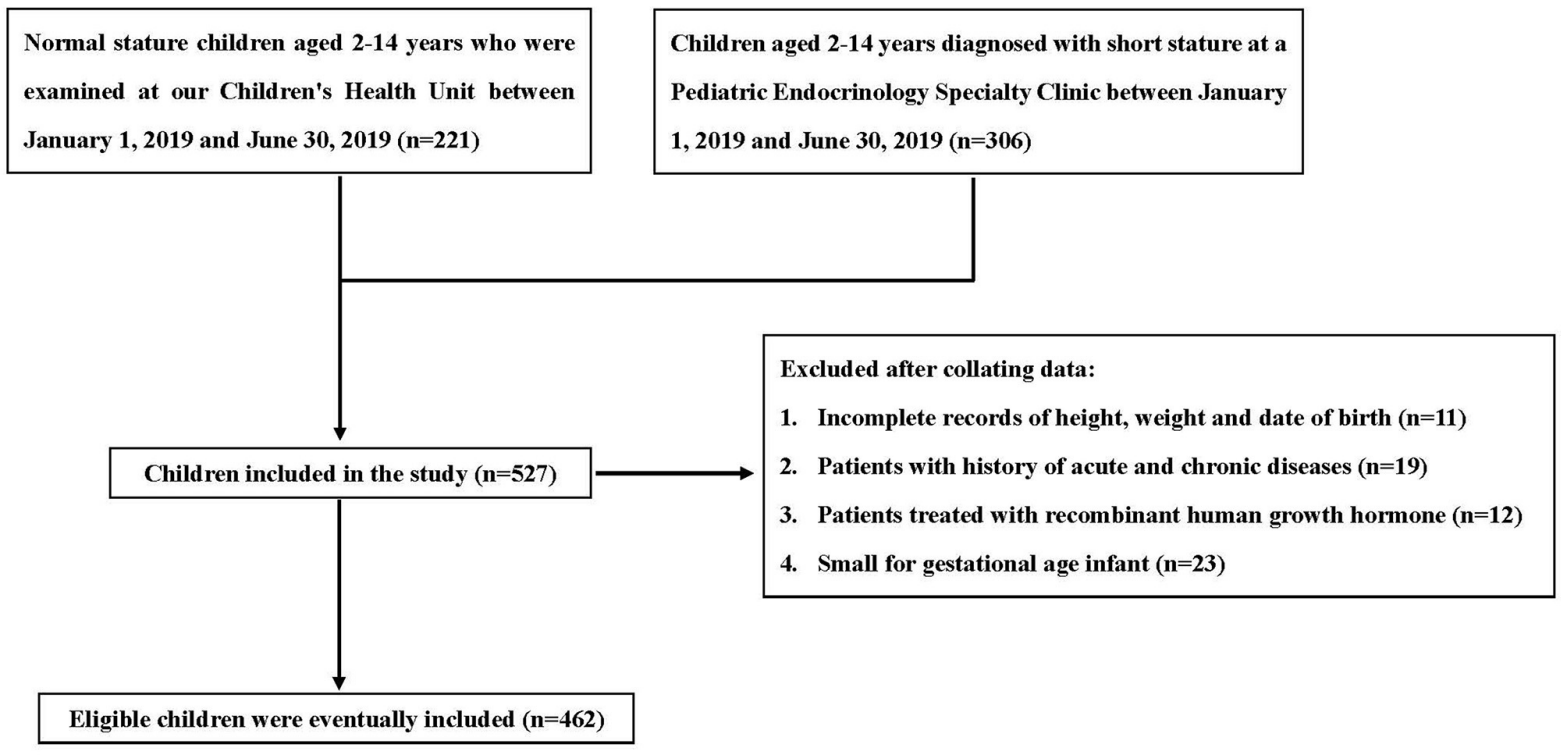
Inclusion and exclusion criteria.

**Figure 2:**
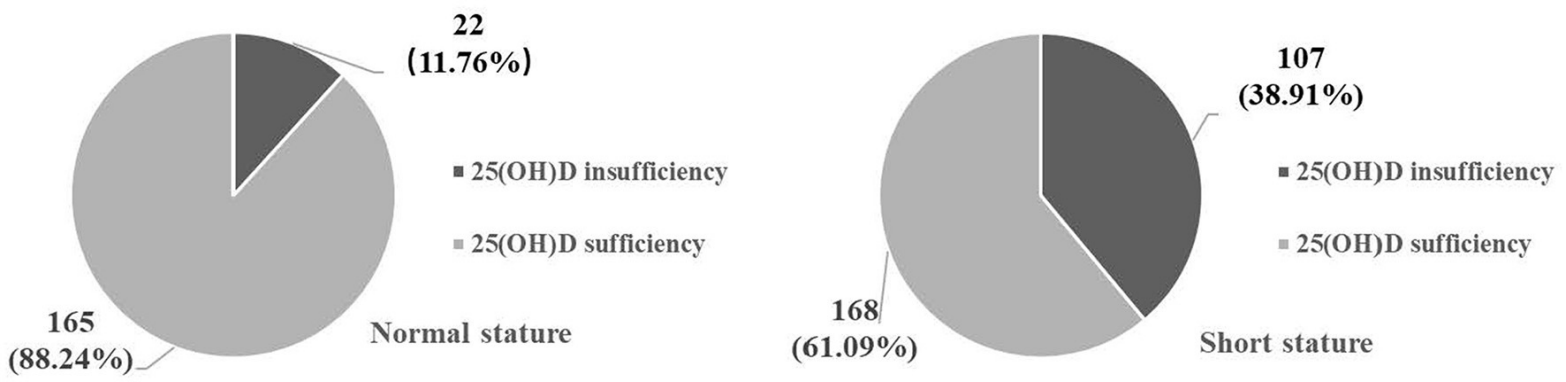
Composition of Serum 25(OH)D levels in different groups [*n* (%)].

A total of 462 participants were selected for the final data analysis through screening (Figure 1). The mean age of the 462 children was 9.76 ± 3.10 years, and 52.16% were male. A total of 129 (27.92%) children were defined as 25(OH)D insufficiency, including 107 (38.91%) in the short stature group and 22 (11.76%) in the normal stature group (Figure 2).

We present the basic characteristics of these selected participants by whether they were short in stature in Table 1. We found statistical differences in age, height, height SDS, weight, BMI, 25(OH)D, sequential day of the year, and birth season between the two groups (all *P* < 0.05). Serum 25(OH)D levels were significantly lower in the short than in the normal stature group (*P* < 0.01). No statistical difference was found between the two groups for gender (*P* > 0.05).

**Table 1:** The basic characteristics of short stature group compared with normal stature group [*n* = 462, Mean ± SD, *n* (%)].

**Group**	**Age (years)**	**Gender**		**Height (cm)**	**Height SDS**	**Weight (kg)**	**BMI (kg/m^**2**^)**	**25(OH)D (ng/ml)**	**Sequential day of the year**	**Season**			
		**Male**	**Female**							**Spring**	**Summer**	**Autumn**	**Winter**
Normal stature (*n* = 187)	10.56 ± 2.76	96 (51.34)	91 (48.66)	139.00 ± 16.76	0.91 ± 0.54	35.36 ± 12.61	17.67 ± 3.04	26.75 ± 4.96	218.52 ± 92.67	26 (13.90)	66 (35.29)	64 (34.23)	31 (16.58)
Short stature (*n* = 275)	9.209 ± 3.21	145 (52.73)	130 (47.27)	121.46 ± 18.44	−2.26 ± 0.19	25.44 ± 11.53	16.39 ± 3.29	22.03 ± 4.07	195.91± 107.04	59 (21.46)	74 (26.91)	74 (26.91)	68 (24.73)
*P*-value	<0.001	0.769		<0.001	<0.001	<0.001	<0.001	<0.001	0.019	0.009			

We present the basic characteristics of these selected participants by season in Table 2. We found statistical differences in age, gender, height, height SDS, weight, sequential day of the year, and short stature across seasonal groups (*P* < 0.01). Children born in summer and autumn were taller and heavier than those born in winter and spring.

**Table 2:** The basic characteristics of participants by season [*n* = 462, Mean ± SD, *n* (%)].

**Season**	**Spring (*n* = 85)**	**Summer (*n* = 140)**	**Autumn (*n* = 138)**	**Winter (*n* = 99)**	***P*-value**
Age (years)	8.66 ± 2.87	10.29 ± 3.09	10.10 ± 3.05	9.47 ± 3.15	<0.001
Gender					<0.001
Male	33 (38.82)	92 (65.71)	64 (46.38%)	52 (52.53)	
Female	52 (61.18)	48 (34.29)	74 (53.62%)	47 (47.47)	
Height (cm)	121.16 ± 16.87	131.55 ± 20.21	131.51 ± 19.10	126.60 ± 20.60	<0.001
Height SDS	−1.29 ± 1.53	−0.80 ± 1.65	−0.76 ± 1.65	−1.27 ± 1.47	0.013
Weight (kg)	24.75 ± 10.05	31.31 ± 13.24	31.08 ± 13.72	28.60 ± 12.66	<0.001
BMI (kg/m^2^)	16.21 ± 2.87	17.22 ± 3.42	17.01 ± 3.38	16.92 ± 3.07	0.145
25(OH)D(ng/ml)	23.75 ± 4.78	24.30 ± 5.33	23.95 ± 4.91	23.56 ± 4.94	0.696
Sequential day of the year	111.55 ± 36.25	202.07 ± 25.91	293.87 ± 26.15	165.80 ± 160.07	<0.001
Short stature					0.009
No	26 (30.59)	66 (47.14)	64 (46.38)	31 (31.31)	
Yes	59 (69.41)	74 (52.86)	74 (53.62)	68 (68.69)	

We present the results of the univariate analysis in Table 3. Through univariate logistic regression, we found a negative association between serum 25(OH)D levels and short stature in children. Each 1 ng/ml increase in serum 25(OH)D was associated with a 20% reduction in the risk of short stature (*P* < 0.01). The risk of short stature was significantly lower in summer and autumn compared with spring (51%, 49%, both *P* < 0.05), whereas the risk of short stature was not significantly lower in winter compared with spring (3%, *P* > 0.05). We also found a negative association between sequential day of the year and the risk of short stature in children; with the increase of the order of birth dates in the year, the risk of short stature decreased (*P* < 0.05).

**Table 3:** Univariate analysis for children's short stature [*n* = 462, Mean ± SD, *n* (%)].

**Characteristics**	**Statistics**	**OR (95%CI)**	***P*-value**
Age (years)	9.76 ± 3.10	0.86 (0.81, 0.92)	<0.001
**Gender**			
Male	241 (52.16)	1.0	
Female	221 (47.84)	0.95 (0.65, 1.37)	0.769
Height (cm)	128.57 ± 19.75	0.95 (0.94, 0.96)	<0.001
Weight (kg)	29.46 ± 12.93	0.94 (0.92, 0.95)	<0.001
BMI (kg/m^2^)	16.91 ± 3.25	0.88 (0.83, 0.94)	<0.001
25(OH)D(ng/ml)	23.94 ± 5.02	0.80 (0.76, 0.84)	<0.001
**Season**			
Spring	85 (18.40)	1.0	
Summer	140 (30.30)	0.49 (0.28, 0.87)	0.015
Autumn	138 (29.87)	0.51 (0.29, 0.90)	0.020
Winter	99 (21.43)	0.97 (0.52, 1.81)	0.916
Sequential day of the year	205.06 ± 101.97	0.99 (0.99, 0.99)	0.020

*Abbreviations: CI, confidence interval. OR, odds ratio*.

*Data in the table: OR (95%CI), p-value*.

*Result variable: Short stature*.

*Exposure variable: Age, Gender, Height, Weight, BMI, 25(OH)D, Season, Sequential day of the year*.

*Adjustment variable: None*.

In all three models, those children born in summer had a reduced risk of short stature compared with spring, and this relationship was essentially stable (<10% change in OR across the three models). Children born in autumn had a reduced risk of short stature compared with the spring (*P* = 0.02), but the relationship was no longer stable after adjusting for age, weight, and 25(OH)D (*P* > 0.05). The risk of short stature was unstable in children born in winter compared with spring, and the difference was not statistically significant (*P* > 0.05). Therefore, in the fully adjusted Model III, the risk of short stature was reduced by 56.5% in children born in summer compared with spring (*P* < 0.05) (Table 4).

**Table 4:** The relationship between seasons and short stature in children in different models.

	**OR (95%CI)**			
	**Spring**	**Summer**	**Autumn**	**Winter**
Model I	1.0	0.494 (0.280, 0.872)	0.510 (0.288, 0.901)	0.967 (0.516, 1.810)
*P*-value		0.015	0.020	0.916
Model II	1.0	0.518 (0.280, 0.960)	0.573 (0.312, 1.053)	1.063 (0.544, 2.077)
*P*-value		0.037	0.073	0.858
Model III	1.0	0.435 (0.197, 0.959)	0.537 (0.245, 1.175)	0.921 (0.388, 2.183)
*P*-value		0.039	0.119	0.851

*Data in the table: OR (95%CI), p-value*.

*Result variable: Short stature*.

*Exposure variable: Season*.

*Model I adjust for: None*.

*Model II adjust for: Gender, Age (Smooth)*.

*Model III adjust for: Gender, Age (Smooth), Weight (Smooth), 25(OH)D*.

*Generalized additive models were applied*.

**Figure 3:**
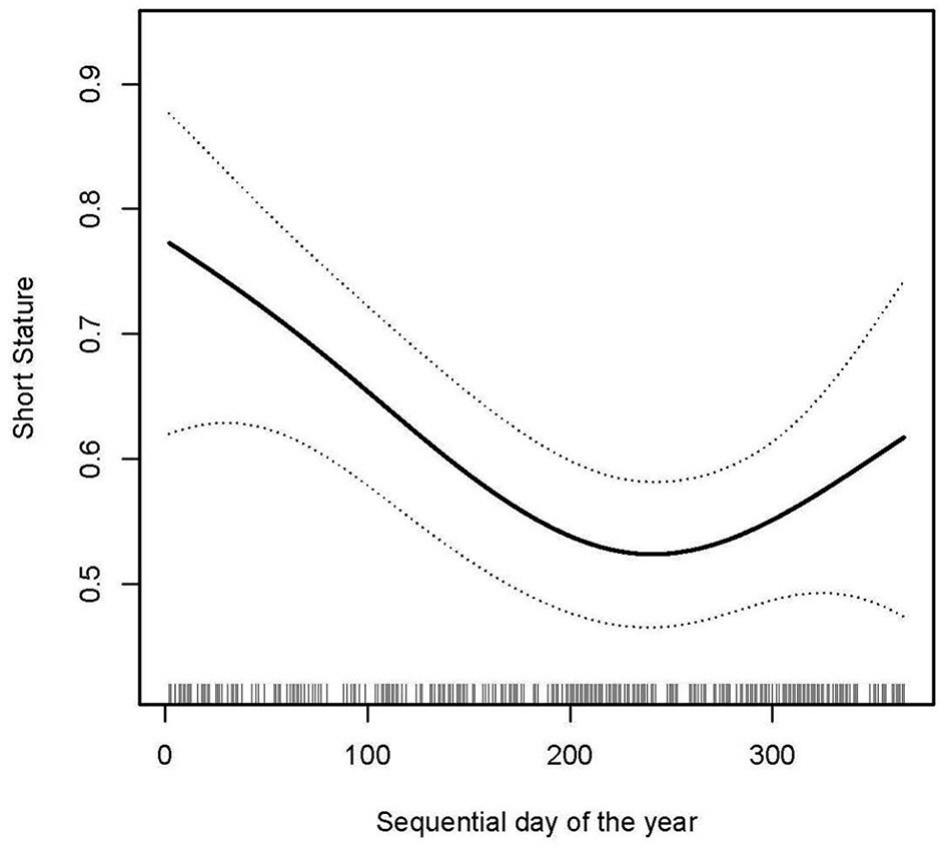
Non-linear relationship between sequential day of the year and short stature (smooth curve fitting). Outcome: Short stature. Exposure: Sequential day of the year. Adjust for gender, age, weight, 25(OH)D.

In the present study, we analyzed the non-linear relationship between sequential day of the year and short stature (Figure 3). A smooth curve and the result of the generalized additive model show that the relationship between sequential day of the year and short stature was non-linear after adjusting for age, gender, weight, and 25(OH)D.

We used both logistic regression and two-piecewise logistic regression to fit the association and select the best fit model based on *P*-values for the log likelihood ratio test. Because the *P*-values for the log likelihood ratio test were <0.05, we chose two-piecewise logistic regression for fitting the association between sequential day of the year and short stature because it could accurately represent the relationship (Table 5). By two-piecewise logistic regression and a recursive algorithm, we calculated the inflection point to be 250. Because of the small effect value, we kept three decimal places. On the left side of the inflection point, the effect size and 95%CI were 0.994, 0.990–0.998, respectively. On the right side of inflection point, the effect size and 95%CI were 1.008, 1.001–1.025, respectively. Which means that from the 1st to the 193rd day of the year, the risk of short stature in children was reduced by 0.6% for each day that passes (*P* = 0.002), and from days 193 to 365 of the year, the risk of short stature in children was increased by 0.8% for each day that passed (*P* = 0.008).

**Table 5:** Threshold effect analysis of sequential day of the year and children's short stature using piece-wise logistic regression.

	**OR (95%CI)**	***P*-value**
**Model I**
One-line slope	0.998 (0.996, 1.000)	0.069
**Model II**
Turning point (K)	250
< K slope 1	0.994 (0.990, 0.998)	0.002
> K slope 2	1.008 (1.001, 1.025)	0.008
LRT test	0.007

*Abbreviations: LRT, Likelihood Ratio Test*.

*Outcome: Short stature*.

*Exposure: Sequential day of the year*.

*Adjust for: Gender, Age, Weight, 25(OH)D*.

## Discussion

Short stature in children has become a hot topic of concern for society and families. Short stature used to be considered an “incurable disease,” but now it is possible to improve short stature through standardized treatment (such as exogenous recombinant human growth hormone supplementation) to achieve a lifetime height that is relatively satisfactory for society and families, which is a great progress in medicine. Even though the pathogenesis of short stature in children is reported in the literature, further research and exploration of possible causative mechanisms bring more benefits to children with short stature.

Several studies find that children's growth and development exhibit distinct seasonal rhythms. Felix et al. (10) find, based on 452,399 population births in relation to season, that those born in summer (June-July-August) had a greater average birth weight, later pubertal development, and more increased adult height compared with those born in other seasons. Lei et al. (12) report the relationship between stunting and season of birth in children under 3 years old in low-income counties in western China and find that children born in summer are less likely to have stunting than those born in winter (OR: 0.74–0.97). Delecroix et al. (13) report that 25(OH)D serum concentrations were lower in cold winter-spring than in warm summer-autumn (31 ± 13 vs. 42 ± 18 ng/ml, *P* = 0.03) and higher in males than in females (37.6 ± 18 vs. 27.6 ± 17.3 ng/ml, *P* = 0.04), which may be related to the seasonal differences of light intensity. Pruszkowska et al. (14) find that individual height might be related to time of birth and season of birth. Adequate sunlight exposure activates 25(OH)D, which is also involved in lipid metabolism, whereas low 25(OH)D levels might increase the risk of obesity. Therefore, the relative lack of 25(OH)D in children born in winter affects bone growth and also indirectly leads to an increase in weight and BMI, and obesity can further contribute to short stature. Sichieri et al. (15) conclude that there is a strong correlation between obesity and short stature after taking into account diet, physical activity, and many environmental factors. The odds of obesity are twice as high in males of short stature as in males of normal stature and, in females, three times as high. When comparing BMI at age 20, the OR was even greater (about six times more in males and eight times more in females). Brabec et al. (16) find that among children born in the same year, those born between February and May (rainy season) were taller than those born between August and November (end of the dry season and beginning of the rainy season). Tanaka et al. (17) find that month and season of birth are thought to affect height, weight, and obesity in school-age children. Children born from May to March of the following year (from late spring to early winter) show a gradual decrease in height and weight, a trend that is repeated every year. Children born in the summer increased in height compared with those born in the fall, and no differences were seen in obesity levels between males and females born in different seasons. In a meta-analysis of 25(OH)D levels during pregnancy and maternal, neonatal, and infant health outcomes, Andrews et al. (18) conclude that 25(OH)D supplementation significantly reduced the risk of low birth weight by 60%. Studies by Bowyer et al. (19) and Leffelaar et al. (20)find that females with 25(OH)D deficiency (25(OH)D thresholds <25 and 29 nmol/l, respectively) gave birth to infants with significantly lower birth weight. Morley et al. (21) find that lower maternal 25(OH)D levels (<28 nmol/l) had no significant effect on mean birth weight, but their offspring had altered 25(OH)D receptor (*Fok*I) genotypes. That is, females with higher 25(OH)D levels had significantly heavier offspring with FF or Ff genotypes, but no weight change was seen in the ff genotype. Brooke et al. (22) report that the offspring of females supplemented with 25(OH)D gained weight faster and were taller than normal females without 25(OH)D supplementation.

In the present study, we find a possible linear and non-linear relationship between different birth seasons and short stature in children. Children born in summer have a significantly lower risk of short stature than children born in spring. The risk rate of short stature in children also tends to decrease with increasing sequential day of the year and begins to show an increasing trend after the 250th day of the year, and this change in pattern is similar to the change of seasons. Based on the results of this study, we hypothesize that the reason for the association of short stature and the season of birth is due to the intensity and duration of sunlight being greater in summer than in spring. This leads to a relative deficit in the amount of 25(OH)D obtained during the late fetal period and after the birth of the infant. The level of 25(OH)D obtained clinically from breast milk alone is relatively low and does not fully meet the growth and developmental needs of the child after birth. Therefore, we recommend that pregnant mothers and postnatal infants take adequate intensity and sufficient hours of sunlight and obtain adequate amounts of exogenous 25(OH)D supplementation. These may go some way to reducing the occurrence of short stature in children. The results of this study can be useful when pregnant mothers come to the hospital for physical examinations or newborn care, which may be an inexpensive and efficient way for prevention. Our results will be useful in further enlightening research on environmental factors associated with the season of birth that contribute to disease in adulthood. This study may help parents who are concerned about this issue to prepare for pregnancy and enhance nutritional support for newborns to reduce the incidence of this disease.

## Conclusions

Children born in summer have a lower risk of short stature than spring. For children born before the 250th day of the year, sequential day of the year is negatively associated with short stature, and for children born after the 250th day, sequential day of the year is positively associated with short stature.

## Strengths and Limitations

Our research has some advantages. (1) We use multiple logistic regression to quantify the independent role between independent and dependent variables; (2) we solve the non-linear problem in this study and further explored this point; (3) we are the first to describe the association between birth time rhythm and short stature of 2- to 14-year-old children in Changde city.

There are some limitations in this study: (1) This study focuses on children in Changde. Therefore, there are some defects in the universality and inference of the research. (2) Because we exclude children with a history of acute or chronic medical conditions, small for gestational age, or children who have received recombinant human growth hormone therapy, the results of this study cannot be applied to these individuals. (3) Although all children in this study were randomly included and not selected on the basis of serum 25(OH)D levels, the fact that all children in the short stature group were from a Pediatric Endocrinology Specialty Clinic may result in some selection bias, which possibly limits the application of study population.

## Data Availability Statement

The data analyzed in this study is subject to the following licenses/restrictions: The raw data supporting the conclusions of this article will be made available by the authors, without undue reservation. Requests to access these datasets should be directed to Cheng Wang, wangcheng2nd@csu.edu.cn.

## Ethics Statement

The studies involving human participants were reviewed and approved by the Ethics Committee of the First People's Hospital of Changde City. Written informed consent to participate in this study was provided by the participants' legal guardian/next of kin.

## Author Contributions

SW had primary responsibility for the protocol development, patient enrollment. SW, NS, and YD had collected data. SW had finished preliminary data analysis and written the manuscript. CW, HC, and RZ assisted with data analysis and critical revision for important content. All authors have read and approved the final manuscript and assumed full responsibility for its contents.

## Funding

This work was supported by grants from 2020 Hunan Province Clinical Medical Technology Innovation Guidance Project (2020SK53405).

## Conflict of Interest

The authors declare that the research was conducted in the absence of any commercial or financial relationships that could be construed as a potential conflict of interest.

## Publisher's Note

All claims expressed in this article are solely those of the authors and do not necessarily represent those of their affiliated organizations, or those of the publisher, the editors and the reviewers. Any product that may be evaluated in this article, or claim that may be made by its manufacturer, is not guaranteed or endorsed by the publisher.
